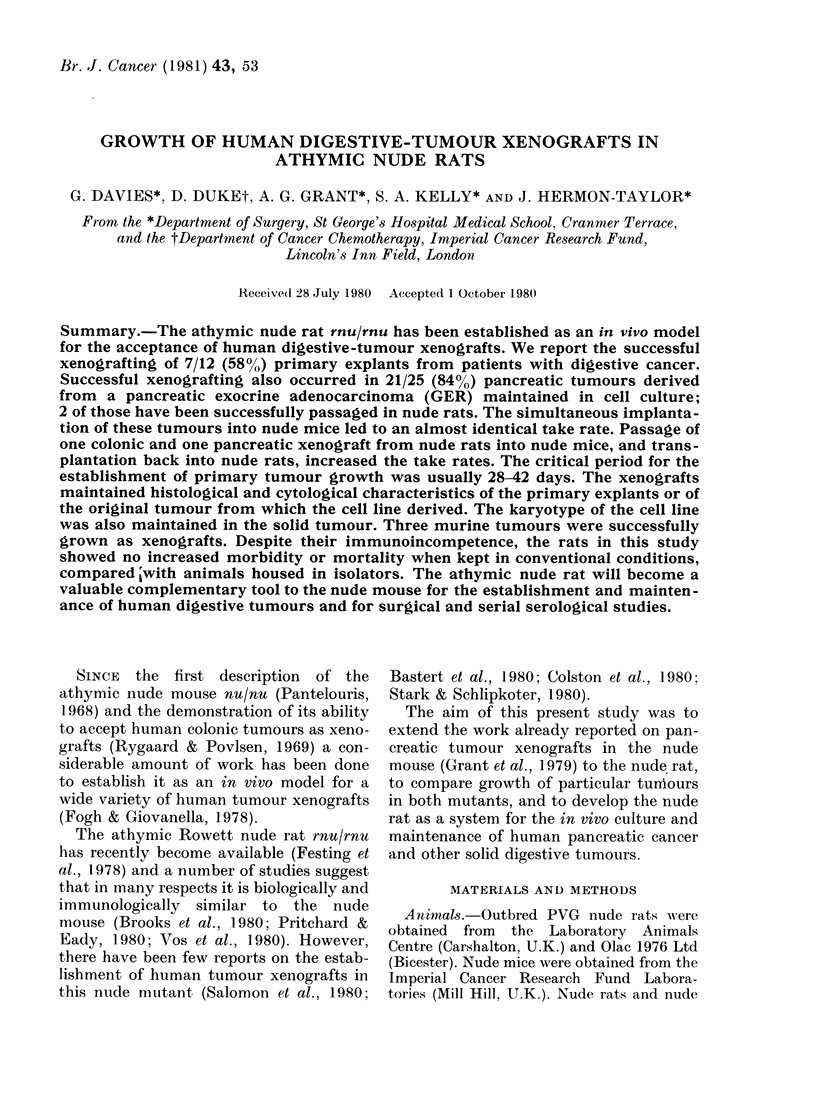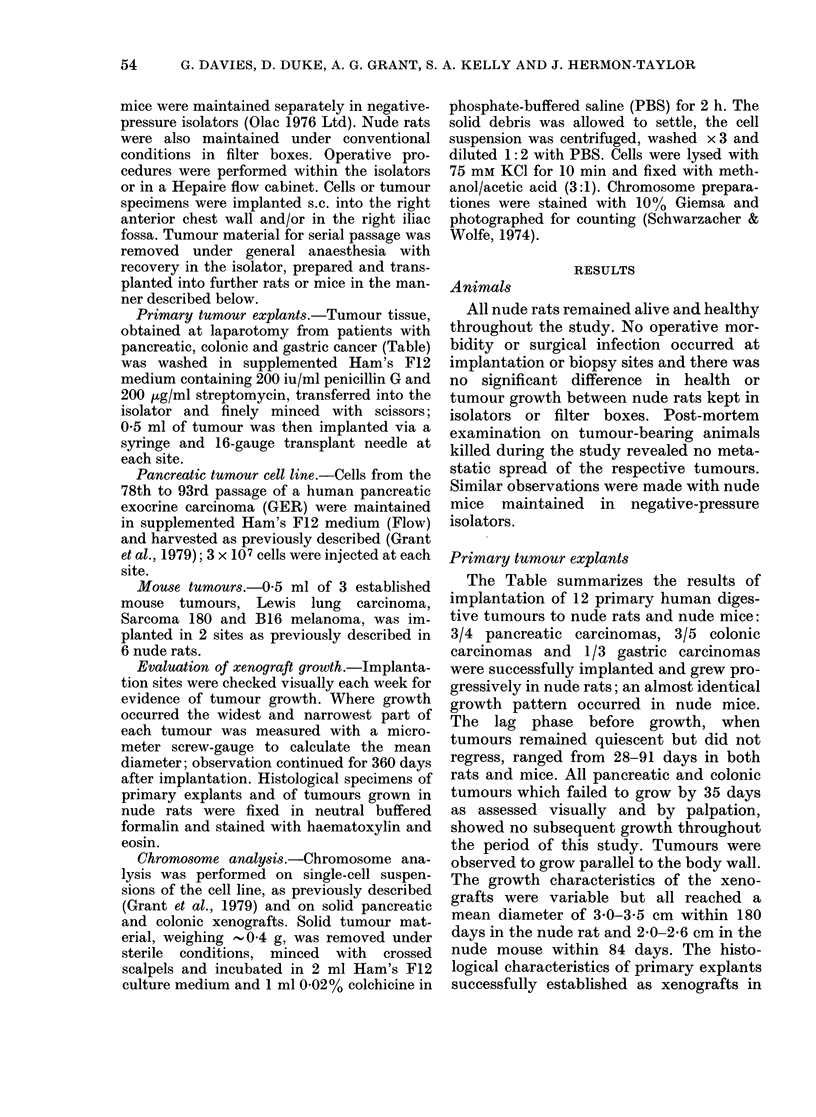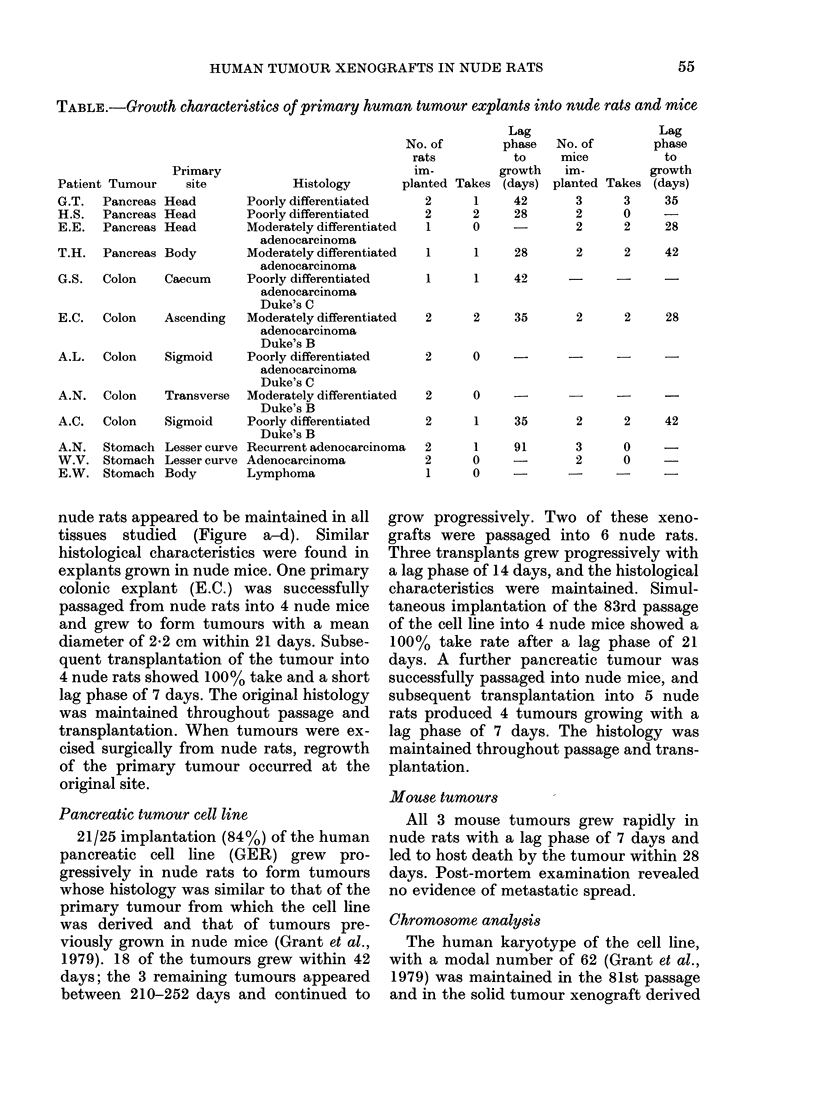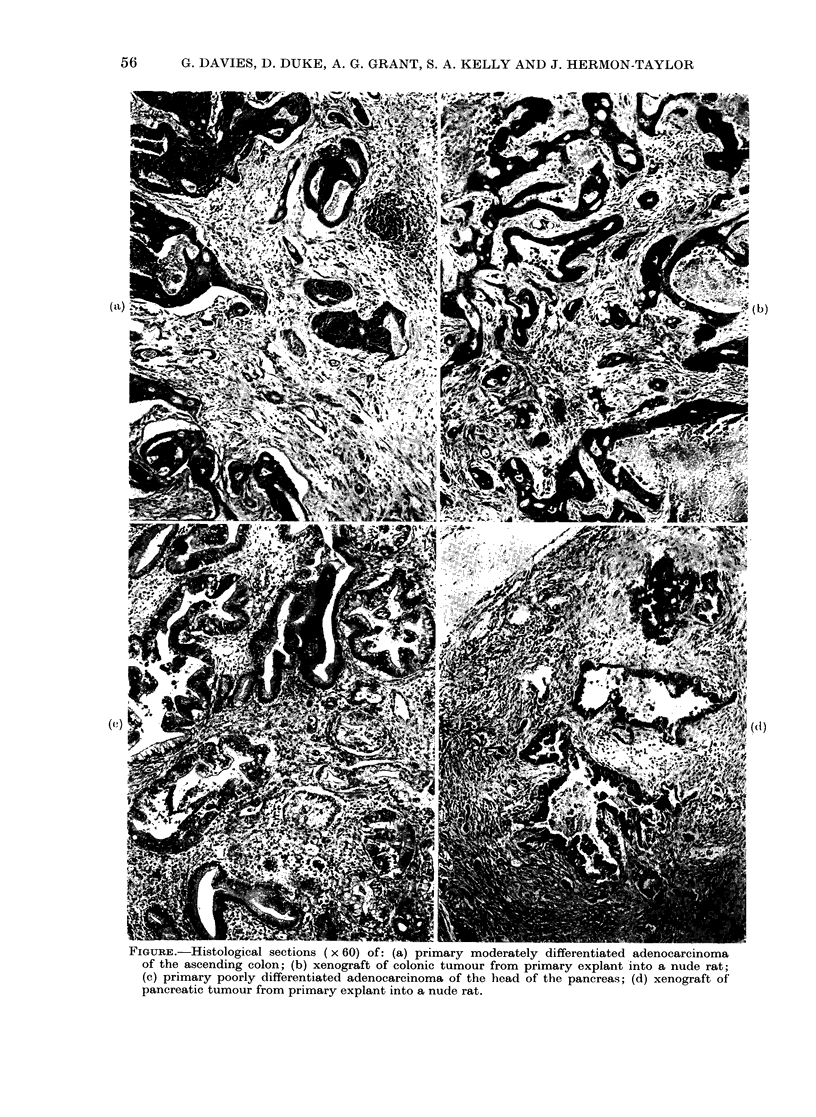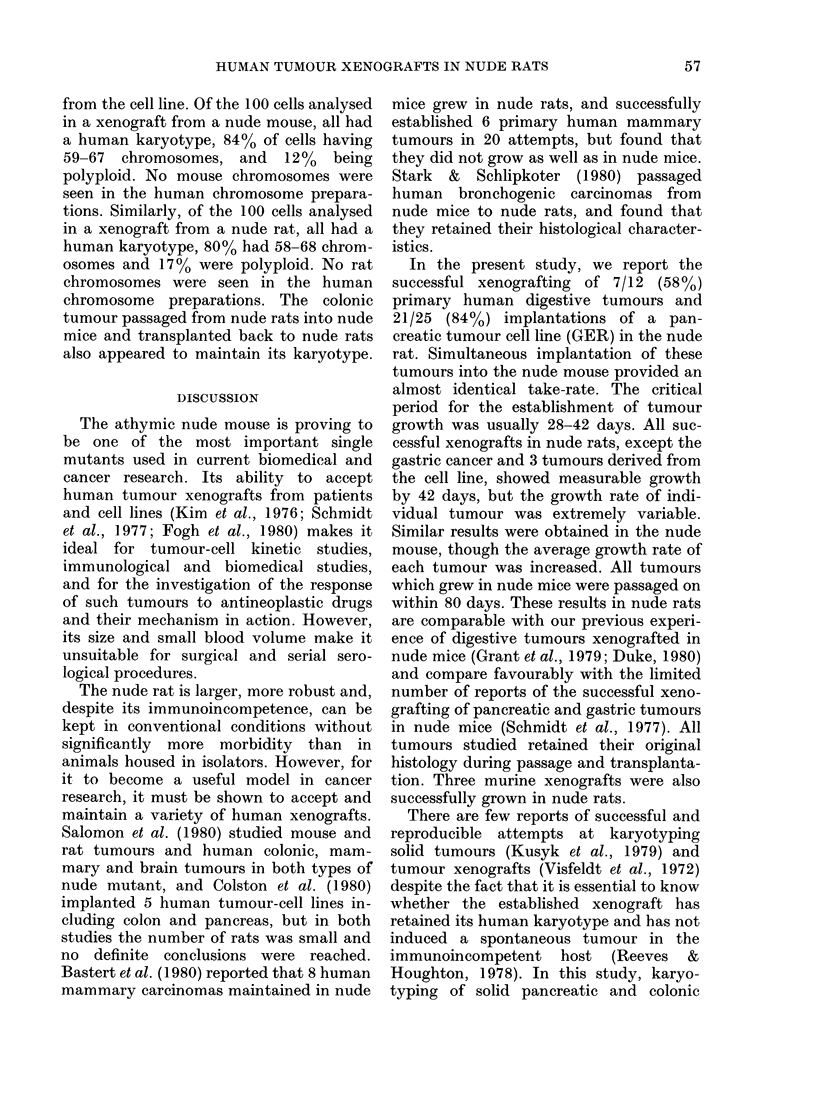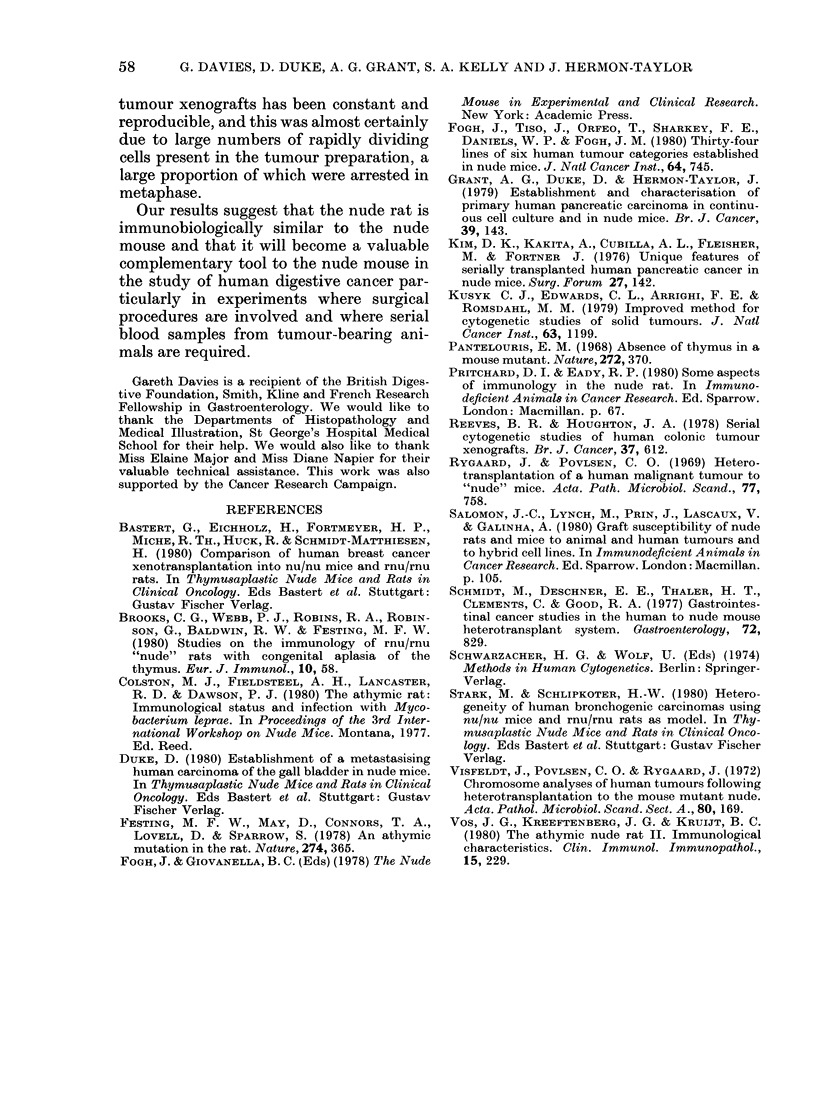# Growth of human digestive-tumour xenografts in athymic nude rats.

**DOI:** 10.1038/bjc.1981.7

**Published:** 1981-01

**Authors:** G. Davies, D. Duke, A. G. Grant, S. A. Kelly, J. Hermon-Taylor

## Abstract

**Images:**


Br. J. Cancer (1981) 43, 53

### GROWTH OF HUMAN DIGESTIVE-TUMOUR XENOGRAFTS IN ATHYMIC NUDE RATS

G. DAVIES*, D. DUKE†, A. G. GRANT*, S. A. KELLY* AND J. HERMON-TAYLOR*

*From the *Department of Surgery, St George's Hospital Medical School, Cranmer Terrace, and the †Department of Cancer Chemotherapy, Imperial Cancer Research Fund, Lincoln's Inn Field, London*

Received 28 July 1980 Accepted 1 October 1980

**Summary.—The athymic nude rat *rnu/rnu* has been established as an *in vivo* model for the acceptance of human digestive-tumour xenografts. We report the successful xenografting of 7/12 (58%) primary explants from patients with digestive cancer. Successful xenografting also occurred in 21/25 (84%) pancreatic tumours derived from a pancreatic exocrine adenocarcinoma (GER) maintained in cell culture; 2 of those have been successfully passaged in nude rats. The simultaneous implantation of these tumours into nude mice led to an almost identical take rate. Passage of one colonic and one pancreatic xenograft from nude rats into nude mice, and transplantation back into nude rats, increased the take rates. The critical period for the establishment of primary tumour growth was usually 28–42 days. The xenografts maintained histological and cytological characteristics of the primary explants or of the original tumour from which the cell line derived. The karyotype of the cell line was also maintained in the solid tumour. Three murine tumours were successfully grown as xenografts. Despite their immunoincompetence, the rats in this study showed no increased morbidity or mortality when kept in conventional conditions, compared with animals housed in isolators. The athymic nude rat will become a valuable complementary tool to the nude mouse for the establishment and maintenance of human digestive tumours and for surgical and serial serological studies.**

SINCE the first description of the athymic nude mouse *nu/nu* (Pantelouris, 1968) and the demonstration of its ability to accept human colonic tumours as xenografts (Rygaard & Povlsen, 1969) a considerable amount of work has been done to establish it as an *in vivo* model for a wide variety of human tumour xenografts (Fogh & Giovanella, 1978).

The athymic Rowett nude rat *rnu/rnu* has recently become available (Festing *et al.*, 1978) and a number of studies suggest that in many respects it is biologically and immunologically similar to the nude mouse (Brooks *et al.*, 1980; Pritchard & Eady, 1980; Vos *et al.*, 1980). However, there have been few reports on the establishment of human tumour xenografts in this nude mutant (Salomon *et al.*, 1980; Bastert *et al.*, 1980; Colston *et al.*, 1980; Stark & Schlipkoter, 1980).

The aim of this present study was to extend the work already reported on pancreatic tumour xenografts in the nude mouse (Grant *et al.*, 1979) to the nude rat, to compare growth of particular tumours in both mutants, and to develop the nude rat as a system for the *in vivo* culture and maintenance of human pancreatic cancer and other solid digestive tumours.

### MATERIALS AND METHODS

*Animals.*—Outbred PVG nude rats were obtained from the Laboratory Animals Centre (Carshalton, U.K.) and Olac 1976 Ltd (Bicester). Nude mice were obtained from the Imperial Cancer Research Fund Laboratories (Mill Hill, U.K.). Nude rats and nude mice were maintained separately in negative-pressure isolators (Olac 1976 Ltd). Nude rats were also maintained under conventional conditions in filter boxes. Operative procedures were performed within the isolators or in a Hepaire flow cabinet. Cells or tumour specimens were implanted s.c. into the right anterior chest wall and/or in the right iliac fossa. Tumour material for serial passage was removed under general anaesthesia with recovery in the isolator, prepared and transplanted into further rats or mice in the manner described below.

*Primary tumour explants.*—Tumour tissue, obtained at laparotomy from patients with pancreatic, colonic and gastric cancer (Table) was washed in supplemented Ham's F12 medium containing 200 iu/ml penicillin G and 200 μg/ml streptomycin, transferred into the isolator and finely minced with scissors; 0·5 ml of tumour was then implanted via a syringe and 16-gauge transplant needle at each site.

*Pancreatic tumour cell line.*—Cells from the 78th to 93rd passage of a human pancreatic exocrine carcinoma (GER) were maintained in supplemented Ham's F12 medium (Flow) and harvested as previously described (Grant *et al.*, 1979); $3 \times 10^7$ cells were injected at each site.

*Mouse tumours.*—0·5 ml of 3 established mouse tumours, Lewis lung carcinoma, Sarcoma 180 and B16 melanoma, was implanted in 2 sites as previously described in 6 nude rats.

*Evaluation of xenograft growth.*—Implantation sites were checked visually each week for evidence of tumour growth. Where growth occurred the widest and narrowest part of each tumour was measured with a micrometer screw-gauge to calculate the mean diameter; observation continued for 360 days after implantation. Histological specimens of primary explants and of tumours grown in nude rats were fixed in neutral buffered formalin and stained with haematoxylin and eosin.

*Chromosome analysis.*—Chromosome analysis was performed on single-cell suspensions of the cell line, as previously described (Grant *et al.*, 1979) and on solid pancreatic and colonic xenografts. Solid tumour material, weighing ~0·4 g, was removed under sterile conditions, minced with crossed scalpels and incubated in 2 ml Ham's F12 culture medium and 1 ml 0·02% colchicine in phosphate-buffered saline (PBS) for 2 h. The solid debris was allowed to settle, the cell suspension was centrifuged, washed ×3 and diluted 1:2 with PBS. Cells were lysed with 75 mM KCl for 10 min and fixed with methanol/acetic acid (3:1). Chromosome preparationes were stained with 10% Giemsa and photographed for counting (Schwarzacher & Wolfe, 1974).

### RESULTS

### *Animals*

All nude rats remained alive and healthy throughout the study. No operative morbidity or surgical infection occurred at implantation or biopsy sites and there was no significant difference in health or tumour growth between nude rats kept in isolators or filter boxes. Post-mortem examination on tumour-bearing animals killed during the study revealed no metastatic spread of the respective tumours. Similar observations were made with nude mice maintained in negative-pressure isolators.

### *Primary tumour explants*

The Table summarizes the results of implantation of 12 primary human digestive tumours to nude rats and nude mice: 3/4 pancreatic carcinomas, 3/5 colonic carcinomas and 1/3 gastric carcinomas were successfully implanted and grew progressively in nude rats; an almost identical growth pattern occurred in nude mice. The lag phase before growth, when tumours remained quiescent but did not regress, ranged from 28–91 days in both rats and mice. All pancreatic and colonic tumours which failed to grow by 35 days as assessed visually and by palpation, showed no subsequent growth throughout the period of this study. Tumours were observed to grow parallel to the body wall. The growth characteristics of the xenografts were variable but all reached a mean diameter of 3·0–3·5 cm within 180 days in the nude rat and 2·0–2·6 cm in the nude mouse within 84 days. The histological characteristics of primary explants successfully established as xenografts in

TABLE.—*Growth characteristics of primary human tumour explants into nude rats and mice*

| Patient | Tumour | Primary site | Histology | No. of rats im-planted | Takes | Lag phase to growth (days) | No. of mice im-planted | Takes | Lag phase to growth (days) |
|---|---|---|---|---|---|---|---|---|---|
| G.T. | Pancreas | Head | Poorly differentiated | 2 | 1 | 42 | 3 | 3 | 35 |
| H.S. | Pancreas | Head | Poorly differentiated | 2 | 2 | 28 | 2 | 0 | — |
| E.E. | Pancreas | Head | Moderately differentiated adenocarcinoma | 1 | 0 | — | 2 | 2 | 28 |
| T.H. | Pancreas | Body | Moderately differentiated adenocarcinoma | 1 | 1 | 28 | 2 | 2 | 42 |
| G.S. | Colon | Caecum | Poorly differentiated adenocarcinoma Duke's C | 1 | 1 | 42 | — | — | — |
| E.C. | Colon | Ascending | Moderately differentiated adenocarcinoma Duke's B | 2 | 2 | 35 | 2 | 2 | 28 |
| A.L. | Colon | Sigmoid | Poorly differentiated adenocarcinoma Duke's C | 2 | 0 | — | — | — | — |
| A.N. | Colon | Transverse | Moderately differentiated Duke's B | 2 | 0 | — | — | — | — |
| A.C. | Colon | Sigmoid | Poorly differentiated Duke's B | 2 | 1 | 35 | 2 | 2 | 42 |
| A.N. | Stomach | Lesser curve | Recurrent adenocarcinoma | 2 | 1 | 91 | 3 | 0 | — |
| W.V. | Stomach | Lesser curve | Adenocarcinoma | 2 | 0 | — | 2 | 0 | — |
| E.W. | Stomach | Body | Lymphoma | 1 | 0 | — | — | — | — |

nude rats appeared to be maintained in all tissues studied (Figure a–d). Similar histological characteristics were found in explants grown in nude mice. One primary colonic explant (E.C.) was successfully passaged from nude rats into 4 nude mice and grew to form tumours with a mean diameter of 2·2 cm within 21 days. Subsequent transplantation of the tumour into 4 nude rats showed 100% take and a short lag phase of 7 days. The original histology was maintained throughout passage and transplantation. When tumours were excised surgically from nude rats, regrowth of the primary tumour occurred at the original site.

*Pancreatic tumour cell line*

21/25 implantation (84%) of the human pancreatic cell line (GER) grew progressively in nude rats to form tumours whose histology was similar to that of the primary tumour from which the cell line was derived and that of tumours previously grown in nude mice (Grant *et al.*, 1979). 18 of the tumours grew within 42 days; the 3 remaining tumours appeared between 210–252 days and continued to grow progressively. Two of these xenografts were passaged into 6 nude rats. Three transplants grew progressively with a lag phase of 14 days, and the histological characteristics were maintained. Simultaneous implantation of the 83rd passage of the cell line into 4 nude mice showed a 100% take rate after a lag phase of 21 days. A further pancreatic tumour was successfully passaged into nude mice, and subsequent transplantation into 5 nude rats produced 4 tumours growing with a lag phase of 7 days. The histology was maintained throughout passage and transplantation.

*Mouse tumours*

All 3 mouse tumours grew rapidly in nude rats with a lag phase of 7 days and led to host death by the tumour within 28 days. Post-mortem examination revealed no evidence of metastatic spread.

*Chromosome analysis*

The human karyotype of the cell line, with a modal number of 62 (Grant *et al.*, 1979) was maintained in the 81st passage and in the solid tumour xenograft derived

FIGURE.—Histological sections (×60) of: (a) primary moderately differentiated adenocarcinoma of the ascending colon; (b) xenograft of colonic tumour from primary explant into a nude rat; (c) primary poorly differentiated adenocarcinoma of the head of the pancreas; (d) xenograft of pancreatic tumour from primary explant into a nude rat.

from the cell line. Of the 100 cells analysed in a xenograft from a nude mouse, all had a human karyotype, 84% of cells having 59–67 chromosomes, and 12% being polyploid. No mouse chromosomes were seen in the human chromosome preparations. Similarly, of the 100 cells analysed in a xenograft from a nude rat, all had a human karyotype, 80% had 58–68 chromosomes and 17% were polyploid. No rat chromosomes were seen in the human chromosome preparations. The colonic tumour passaged from nude rats into nude mice and transplanted back to nude rats also appeared to maintain its karyotype.

### DISCUSSION

The athymic nude mouse is proving to be one of the most important single mutants used in current biomedical and cancer research. Its ability to accept human tumour xenografts from patients and cell lines (Kim *et al.*, 1976; Schmidt *et al.*, 1977; Fogh *et al.*, 1980) makes it ideal for tumour-cell kinetic studies, immunological and biomedical studies, and for the investigation of the response of such tumours to antineoplastic drugs and their mechanism in action. However, its size and small blood volume make it unsuitable for surgical and serial serological procedures.

The nude rat is larger, more robust and, despite its immunoincompetence, can be kept in conventional conditions without significantly more morbidity than in animals housed in isolators. However, for it to become a useful model in cancer research, it must be shown to accept and maintain a variety of human xenografts. Salomon *et al.* (1980) studied mouse and rat tumours and human colonic, mammary and brain tumours in both types of nude mutant, and Colston *et al.* (1980) implanted 5 human tumour-cell lines including colon and pancreas, but in both studies the number of rats was small and no definite conclusions were reached. Bastert *et al.* (1980) reported that 8 human mammary carcinomas maintained in nude mice grew in nude rats, and successfully established 6 primary human mammary tumours in 20 attempts, but found that they did not grow as well as in nude mice. Stark & Schlipkoter (1980) passaged human bronchogenic carcinomas from nude mice to nude rats, and found that they retained their histological characteristics.

In the present study, we report the successful xenografting of 7/12 (58%) primary human digestive tumours and 21/25 (84%) implantations of a pancreatic tumour cell line (GER) in the nude rat. Simultaneous implantation of these tumours into the nude mouse provided an almost identical take-rate. The critical period for the establishment of tumour growth was usually 28–42 days. All successful xenografts in nude rats, except the gastric cancer and 3 tumours derived from the cell line, showed measurable growth by 42 days, but the growth rate of individual tumour was extremely variable. Similar results were obtained in the nude mouse, though the average growth rate of each tumour was increased. All tumours which grew in nude mice were passaged on within 80 days. These results in nude rats are comparable with our previous experience of digestive tumours xenografted in nude mice (Grant *et al.*, 1979; Duke, 1980) and compare favourably with the limited number of reports of the successful xenografting of pancreatic and gastric tumours in nude mice (Schmidt *et al.*, 1977). All tumours studied retained their original histology during passage and transplantation. Three murine xenografts were also successfully grown in nude rats.

There are few reports of successful and reproducible attempts at karyotyping solid tumours (Kusyk *et al.*, 1979) and tumour xenografts (Visfeldt *et al.*, 1972) despite the fact that it is essential to know whether the established xenograft has retained its human karyotype and has not induced a spontaneous tumour in the immunoincompetent host (Reeves & Houghton, 1978). In this study, karyotyping of solid pancreatic and colonic tumour xenografts has been constant and reproducible, and this was almost certainly due to large numbers of rapidly dividing cells present in the tumour preparation, a large proportion of which were arrested in metaphase.

Our results suggest that the nude rat is immunobiologically similar to the nude mouse and that it will become a valuable complementary tool to the nude mouse in the study of human digestive cancer particularly in experiments where surgical procedures are involved and where serial blood samples from tumour-bearing animals are required.

Gareth Davies is a recipient of the British Digestive Foundation, Smith, Kline and French Research Fellowship in Gastroenterology. We would like to thank the Departments of Histopathology and Medical Illustration, St George's Hospital Medical School for their help. We would also like to thank Miss Elaine Major and Miss Diane Napier for their valuable technical assistance. This work was also supported by the Cancer Research Campaign.

### REFERENCES

BASTERT, G., EICHHOLZ, H., FORTMEYER, H. P., MICHE, R. TH., HUCK, R. & SCHMIDT-MATTHIESEN, H. (1980) Comparison of human breast cancer xenotransplantation into nu/nu mice and rnu/rnu rats. In *Thymusaplastic Nude Mice and Rats in Clinical Oncology*. Eds Bastert *et al.* Stuttgart: Gustav Fischer Verlag.

BROOKS, C. G., WEBB, P. J., ROBINS, R. A., ROBINSON, G., BALDWIN, R. W. & FESTING, M. F. W. (1980) Studies on the immunology of rnu/rnu "nude" rats with congenital aplasia of the thymus. *Eur. J. Immunol.*, **10**, 58.

COLSTON, M. J., FIELDSTEEL, A. H., LANCASTER, R. D. & DAWSON, P. J. (1980) The athymic rat: Immunological status and infection with *Mycobacterium leprae*. In *Proceedings of the 3rd International Workshop on Nude Mice*. Montana, 1977. Ed. Reed.

DUKE, D. (1980) Establishment of a metastasising human carcinoma of the gall bladder in nude mice. In *Thymusaplastic Nude Mice and Rats in Clinical Oncology*. Eds Bastert *et al.* Stuttgart: Gustav Fischer Verlag.

FESTING, M. F. W., MAY, D., CONNORS, T. A., LOVELL, D. & SPARROW, S. (1978) An athymic mutation in the rat. *Nature*, **274**, 365.

FOGH, J. & GIOVANELLA, B. C. (Eds) (1978) *The Nude Mouse in Experimental and Clinical Research.* New York: Academic Press.

FOGH, J., TISO, J., ORFEO, T., SHARKEY, F. E., DANIELS, W. P. & FOGH, J. M. (1980) Thirty-four lines of six human tumour categories established in nude mice. *J. Natl Cancer Inst.*, **64**, 745.

GRANT, A. G., DUKE, D. & HERMON-TAYLOR, J. (1979) Establishment and characterisation of primary human pancreatic carcinoma in continuous cell culture and in nude mice. *Br. J. Cancer*, **39**, 143.

KIM, D. K., KAKITA, A., CUBILLA, A. L., FLEISHER, M. & FORTNER J. (1976) Unique features of serially transplanted human pancreatic cancer in nude mice. *Surg. Forum* **27**, 142.

KUSYK C. J., EDWARDS, C. L., ARRIGHI, F. E. & ROMSDAHL, M. M. (1979) Improved method for cytogenetic studies of solid tumours. *J. Natl Cancer Inst.*, **63**, 1199.

PANTELOURIS, E. M. (1968) Absence of thymus in a mouse mutant. *Nature*, **272**, 370.

PRITCHARD, D. I. & EADY, R. P. (1980) Some aspects of immunology in the nude rat. In *Immunodeficient Animals in Cancer Research.* Ed. Sparrow. London: Macmillan. p. 67.

REEVES, B. R. & HOUGHTON, J. A. (1978) Serial cytogenetic studies of human colonic tumour xenografts. *Br. J. Cancer*, **37**, 612.

RYGAARD, J. & POVLSEN, C. O. (1969) Heterotransplantation of a human malignant tumour to "nude" mice. *Acta. Path. Microbiol. Scand.*, **77**, 758.

SALOMON, J.-C., LYNCH, M., PRIN, J., LASCAUX, V. & GALINHA, A. (1980) Graft susceptibility of nude rats and mice to animal and human tumours and to hybrid cell lines. In *Immunodeficient Animals in Cancer Research.* Ed. Sparrow. London: Macmillan. p. 105.

SCHMIDT, M., DESCHNER, E. E., THALER, H. T., CLEMENTS, C. & GOOD, R. A. (1977) Gastrointestinal cancer studies in the human to nude mouse heterotransplant system. *Gastroenterology*, **72**, 829.

SCHWARZACHER, H. G. & WOLF, U. (Eds) (1974) *Methods in Human Cytogenetics*. Berlin: Springer-Verlag.

STARK, M. & SCHLIPKOTER, H.-W. (1980) Heterogeneity of human bronchogenic carcinomas using *nu/nu* mice and rnu/rnu rats as model. In *Thymusaplastic Nude Mice and Rats in Clinical Oncology.* Eds Bastert *et al.* Stuttgart: Gustav Fischer Verlag.

VISFELDT, J., POVLSEN, C. O. & RYGAARD, J. (1972) Chromosome analyses of human tumours following heterotransplantation to the mouse mutant nude. *Acta. Pathol. Microbiol. Scand. Sect. A.*, **80**, 169.

VOS, J. G., KREEFTENBERG, J. G. & KRUIJT, B. C. (1980) The athymic nude rat II. Immunological characteristics. *Clin. Immunol. Immunopathol.*, **15**, 229.